# mHealth Messaging to Motivate Quitline Use and Quitting: Protocol for a Community-Based Randomized Controlled Trial in Rural Vietnam

**DOI:** 10.2196/30947

**Published:** 2021-10-07

**Authors:** Celine Larkin, Jessica Wijesundara, Hoa L Nguyen, Duc Anh Ha, Anh Vuong, Cuong Kieu Nguyen, Daniel Amante, Chau Quy Ngo, Phuong Thu Phan, Quyen Thi Le Pham, Binh Ngoc Nguyen, Anh Thi Phuong Nguyen, Phuong Thi Thu Nguyen, Sharina Person, Jeroan J Allison, Thomas K Houston, Rajani Sadasivam

**Affiliations:** 1 Department of Emergency Medicine University of Massachusetts Medical School Worcester, MA United States; 2 Department of Population and Quantitative Health Sciences University of Massachusetts Medical School Worcester, MA United States; 3 Vietnam Ministry of Health Hanoi Vietnam; 4 Institute of Population, Health and Development Hanoi Vietnam; 5 Respiratory Center Bach Mai Hospital Hanoi Vietnam; 6 Wake Forest School of Medicine Wake Forest University Winston-Salem, NC United States

**Keywords:** tobacco cessation, smoking cessation, mHealth, global health, Vietnam, randomized controlled trial

## Abstract

**Background:**

Tobacco kills more than 8 million people each year, mostly in low- and middle-income countries. In Vietnam, 1 in every 2 male adults smokes tobacco. Vietnam has set up telephone Quitline counseling that is available to all smokers, but it is underused. We previously developed an automated and effective motivational text messaging system to support smoking cessation among US smokers.

**Objective:**

The aim of this study is to adapt the aforementioned system for rural Vietnamese smokers to promote cessation of tobacco use, both directly and by increasing the use of telephone Quitline counseling services and nicotine replacement therapy. Moreover, we seek to enhance research and health service capacity in Vietnam.

**Methods:**

We are testing the effectiveness of our culturally adapted motivational text messaging system by using a community-based randomized controlled trial design (N=600). Participants were randomly allocated to the intervention (regular motivational and assessment text messages) or control condition (assessment text messages only) for a period of 6 months. Trial recruitment took place in four communes in the Hung Yen province in the Red River Delta region of Vietnam. Recruitment events were advertised to the local community, facilitated by community health workers, and occurred in the commune health center. We are assessing the impact of the texting system on 6-month self-reported and biochemically verified smoking cessation, as well as smoking self-efficacy, uptake of the Quitline, and use of nicotine replacement therapy. In addition to conducting the trial, the research team also provided ongoing training and consultation with the Quitline during the study period.

**Results:**

Site preparation, staff training, intervention adaptation, participant recruitment, and baseline data collection were completed. The study was funded in August 2017; it was reviewed and approved by the University of Massachusetts Medical School Institutional Review Board in 2017. Recruitment began in November 2018. A total of 750 participants were recruited from four communes, and 700 (93.3%) participants completed follow-up by March 2021. An analysis of the trial results is in progress; results are expected to be published in late 2022.

**Conclusions:**

This study examines the effectiveness of mobile health interventions for smoking in rural areas in low- and middle-income countries, which can be implemented nationwide if proven effective. In addition, it also facilitates significant collaboration and capacity building among a variety of international partners, including researchers, policy makers, Quitline counselors, and community health workers.

**Trial Registration:**

ClinicalTrials.gov NCT03567993; https://clinicaltrials.gov/ct2/show/NCT03567993.

**International Registered Report Identifier (IRRID):**

DERR1-10.2196/30947

## Introduction

### Background

Tobacco is a leading preventable cause of mortality, killing more than eight million people each year, mostly in low- and middle-income countries [1]. Although effective smoking cessation aids are available [2], the use of pharmacological cessation aids combined with behavioral counseling doubles the chances of successfully achieving long-term cessation [3]. Unfortunately, these treatments are often underutilized, especially in low- and middle-income countries, where the rates of tobacco use are high. In Vietnam, 44% of men and 1% of women smoke [4]. Evidence-based interventions are underused in Vietnam: only 24% of smokers have used nicotine replacement therapy (NRT), patch, or chewing gum; less than 1% have used prescription medication to try and stop smoking; and only 3% of smokers attempting to quit in Vietnam report receiving counseling [5]. Most smokers in rural Vietnam are thinking of quitting but are not ready to quit the next month [5]. In 2015, the Vietnam Ministry of Health established two telephone *Quitlines*, based in northern Vietnam (the Bach Mai Hospital’s Quitline) and southern Vietnam (the Binh Dan Hospital’s Quitline) to engage and motivate Vietnamese individuals to quit smoking cigarettes. However, these services are underused, and interventions are needed to improve the uptake of these Quitlines and improve the quitting rate in Vietnam.

### Objectives

The objectives of this study are as follows:

- To adapt an existing cessation texting system that has proven effective in the United States [6] to the Vietnamese context, encouraging smokers in rural settings of northern Vietnam to accept counseling services from the existing Bach Mai Hospital Quitline and motivating smokers to quit.
- To conduct a randomized controlled trial to test the effectiveness of the adapted motivational text messaging intervention in improving smoking cessation in a sample of 600 rural Vietnamese smokers.
- To implement capacity building activities for staff at the Bach Mai Quitline.

We hypothesize that participants in the intervention arm will have higher rates of cessation and higher smoking self-efficacy and that they will engage more frequently with the Quitline counseling and NRT services. 

## Methods

### Intervention Development and Adaptation

#### Intervention Overview

In this study, participants are randomized to receive either (1) motivation and assessment text messages that encouraged smoking cessation via Quitline service and NRT engagement (intervention group) or (2) assessment text messages only (comparison group). The text message system is intended to encourage the uptake of cessation services and is, hence, an augmentation of, not a replacement for, regular appropriate clinical services.

#### Message Content and Sequencing

Our message database from a prior study in the United States included both expert and peer-written messages. The expert-written motivational messages were iteratively developed through a group review, with content guided by current guidelines and social cognitive theory [7]. The peer messages were advice messages written by smokers to other smokers. The combination of these messages increased smoking cessation rates in our prior US study. Expert-written messages provided important theory- and guideline-based health information to the participants. The peer messages touched on more social aspects (dealing with family and cost) than expert messages and increased longitudinal engagement of the intervention compared with the expert messages [7].

We used a two-step process to develop a similar database for this study in the Vietnamese language. First, we adapted the expert-written motivational messages that were previously developed in English iteratively through a group review, with content guided by current guidelines and social cognitive theory. After the messages were professionally translated to Vietnamese, consultants (former smokers who had used the Bach Mai Quitline services) and professionals reviewed the existing messages and were asked to identify messages that they liked and disliked. Deriving culturally appropriate message content using peer messages was especially important in this study because it was the first time that the intervention had been implemented in Vietnam. Therefore, 10 consultants (former smokers who had used the Quitline in Vietnam) were invited to draft new text messages based on prompts such as “I called the Bach Mai Quitline when...” and “I quit smoking because...” The research team then eliminated messages that were incomplete, unclear, or clearly redundant and established a set of shortlisted messages. This process is outlined in the *Results* section.

In addition to these one-way motivational text messages, the research team developed a series of two-way assessment messages in Vietnamese. All participants (intervention and control arms) received the following text message every two weeks: “How many times have you smoked tobacco in the past 24 hours?” while intervention participants also received a Quitline-related question every other week: “Would you like a call from a Bach Mai Quitline smoking counselor?” If they replied with a “yes,” the Quitline would call them back. The final sequences of the messages were collaboratively reviewed and approved by the research team.

#### Bach Mai Quitline

Quitlines have been demonstrated to be an effective intervention for smoking cessation [8]. Trained counselors can help callers select the appropriate cessation services for them and can offer call-back strategies to maximize adherence to NRT and help with relapse prevention. Interventions to increase the use of Quitlines have included connecting with clinics and email and text motivational reminders [9]. The Bach Mai Quitline in Vietnam is staffed by certified tobacco treatment specialists (registered nurses and public health professionals) who have been trained in principles of motivational interviewing, evidence of the risks associated with tobacco, benefits of phone-based counseling, and strategies summarized in the 2008 US Treating Tobacco Use and Dependence guidelines [10] including the *5As* (Ask, Advise, Assess, Assist, and Arrange) and the *5Rs* (Relevance, Risks, Rewards, Roadblocks, and Repetition). In this study, the team at the University of Massachusetts Medical School (UMMS) conducted site visits and additional training for the Bach Mai Quitline staff. This additional training totaled 10 hours, used a combination of didactics and role plays, and covered topics such as pharmacotherapy, behavioral interventions for smoking cessation, motivational interviewing, individual counseling, telephone counseling, and the association between tobacco use and COVID-19. During the trial, Quitline counselors were able to facilitate NRT for participants at no cost.

### Trial Design

#### Trial Design Overview

**Figure 1:**
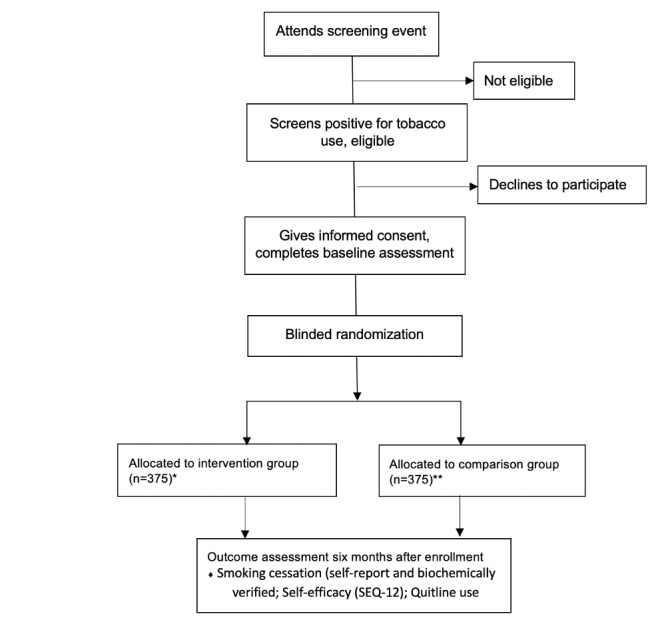
Study scheme. *The intervention group received one-way motivational text messages (seven in the first week, two per week in weeks 2-26) to promote cessation and provide encouragement to access Quitline and free nicotine replacement therapy. They also received weekly two-way assessment text messages asking how many times they had smoked tobacco in the past 24 hours and whether they would like to receive a call from the Quitline, which is then provided. **The control group received twice-monthly two-way assessment text messages asking how many times they have smoked tobacco in the past 24 hours. SEQ-12: 12-item Smoking Self-Efficacy Questionnaire.

The study design is a two-arm randomized controlled trial of 600 participants (300 participants in the intervention group and 300 in the comparison group) from Hung Yen province of Vietnam (Figure 1). The intervention group received one-way motivational text messages (seven in the first week, two per week in weeks 2-26) to promote cessation and provide encouragement to access Quitline and free NRT. They also received weekly two-way assessment text messages asking how many times they had smoked tobacco in the past 24 hours and whether they would like to receive a call from the Quitline, which is then provided. The control group received twice-monthly two-way assessment text messages asking how many times they have smoked tobacco in the past 24 hours. The study is being implemented by the Institute of Population, Health, and Development (PHAD), Hanoi, Vietnam, and is based on a long-term partnership between the Institute and UMMS.

#### Study Setting and Inclusion Criteria

Hung Yen province has a population of about 1.2 million, organized into 10 districts and 161 communes. In Hung Yen province, the vast majority of the residents have a mobile phone. Participants are recruited from four communities (communes) in Hung Yen province: Binh Minh, Viet Hung, Tan Viet, and Bach Sam. Each of the selected communes satisfy the following criteria: (1) have a community health center with a medical doctor; (2) are not currently participating in other studies for smoking cessation; and (3) have a minimum geographic separation of 12 km (7 miles) from all other study communes to minimize possible contamination. To be enrolled in this trial, consenting adult men and women need to fulfill each of the following criteria: (1) be a resident of the selected commune, (2) be a current smoker, (3) be able to receive text and read text (literate), (4) not be cognitively impaired, (5) not be a smoker who helped develop the motivational texts used in the intervention, and (6) not be a family member of another participant in the study. Sex is not an eligibility criterion, but rates of smoking among women are very low in Vietnam; therefore, the sample is likely to be overwhelmingly male.

#### Recruitment Approach

At the beginning of the study, all study personnel and community and Quitline collaborators were trained carefully about the study protocol and standard operational procedures. Advertising of the study is conducted by health workers at community health centers and community health workers. Recruitment events are held at the commune health center on an approximately monthly basis. Interested individuals are screened for eligibility by study staff, who then explain the study to them. If they are eligible and willing to participate, they receive further details about the purpose of the research; procedures involved; their right to decline to participate and withdraw from the research at any time; potential risks, discomfort, or adverse effects; prospective research benefits; incentives for participation; and research participants’ rights. Participants receive either a token of mobile telephone credit for participating or a basic phone that can be used for texting if they do not have a phone. The participants then sign a consent form and proceeded to the baseline assessment and random assignment. Protocol fidelity is determined by the Vietnam coinvestigators who directly monitor 20% (150/750) of all study enrollment and follow-up visits and complete a fidelity checklist.

#### Randomization

All participants are told that they will be randomized to either the intervention or the comparison group. The allocation of participants to study arms is based on a permuted block scheme in which treatment assignments are made within blocks so that the numbers assigned to each treatment arm are equal after a block has been filled. Blocks of various sizes (2, 4, and 6) are used in random order to facilitate allocation concealment. At the end of the baseline assessment survey, the research staff enter the participant ID and participant mobile phone number from the survey into our texting system, which references the next allocation within the table and adapts based on the allocation assignment. Using this technique, both participants and research staff are blinded to the allocation during the initial session. The research staff are blinded to allocation when assessing outcomes.

#### Intervention Condition

Participants who are randomized to the intervention arm receive one motivational text message per day in the first week of the trial and two motivational messages per week for the next 25 weeks. They also receive weekly two-way assessment messages (described above) that ask the participant whether they have smoked recently and if they would like to receive a call from the Quitline. If they say “yes,” the Quitline calls the patient and offers counseling and access to free NRT. Participants who are randomized to an intervention condition receive encouragement (eg, motivational texts) and the participants themselves choose whether and when to engage in some levels of the intervention (Quitline, NRT) either by texting back to request a call or by calling the Quitline directly. The advantages of this encouragement design are that it provides an indication of uptake or participation, allowing variability in uptake of intervention components and providing an assessment of intervention reach and effective dose.

#### Control Condition

Participants who are randomized to the comparison condition receive only a two-way assessment question every two weeks, asking if they have smoked recently. Participants in the comparison group are not specifically being encouraged by the research team to seek Quitline counseling or NRT, but they could proactively seek it from the local clinic or from the Quitline.

### Sample Size and Power

Our calculations are based on several assumptions. We have estimated the control cessation rate to be 10% in the comparison group, and based on our prior similar trials, we have derived a 9% difference in intervention and control. With a sample of 600 participants who are randomized 1:1 to the intervention and comparison conditions, we are able to detect a 10% difference between groups (two-sided chi-square test, α=.05) with 91% power. On the basis of previous studies, we estimate that there will be about 15%-20% attrition. Thus, although we require a sample size of 600 participants to complete a 6-month follow-up, we plan to randomize 750 participants at baseline (Figure 1). We will monitor recruitment and retention and inflate our sample as needed to achieve the resulting sample of 600 participants completing the 6-month follow-up.

### Data Collection

All patient-facing documents are translated into Vietnamese by a certified and qualified translator. All translators are native speakers of Vietnamese and members of the American Translators Association. Once translated, the documents are edited by an accredited second language expert for readability, terminology, and accuracy. The materials are then proofed for completeness, formatting, and layout. All translated materials receive a certification of accuracy from the translation company.

Baseline data are collected by the research staff during commune-based enrollment events. Baseline data collection includes patient demographics, comorbidities, technology use, and Quitline use. Participants are asked about their level of readiness to quit (response options: not thinking about quitting, thinking about quitting, setting a quit date, quitting smoking today, and having already quit smoking), former quit attempts, and current smoking habits (how many cigarettes smoked per day, other tobacco products used, how soon after they smoke, how old they were when they first smoked, and how many years they smoked every day). Participants are asked how many of their immediate family members, extended family members, close friends, friends, acquaintances, and coworkers smoke. Social support for quitting is measured using the shortened partner interaction questionnaire [11] for those who are married or partnered. Smoking cravings are assessed using the brief 10-item version [12] of the Questionnaire of Smoking Urges [13], which retains the two-factor structure of the original and has high internal consistency [12]. At baseline, we also administer the 12-item Smoking Self-Efficacy Questionnaire (SEQ-12) [14], a questionnaire with two subscales measuring confidence in the ability to refrain from smoking when facing internal and external stimuli, respectively. The scale has high test-retest reliability and is predictive of future cessation, and the subscales have good internal consistency [14].

### Study Outcomes

The primary outcome of the study is the tobacco cessation rate at 6-month follow-up. At the follow-up research visit, the research staff determines current smoking through self-report based on the following question, “Do you currently smoke tobacco (smoked even 1 puff of tobacco in the last 7 days)?” (yes or no), and then verify with a carbon monoxide breath monitor. Patients who respond “no” to smoking are now classified as nonsmokers if their carbon monoxide measurement is less than 10 ppm [15]. Secondary outcomes are changes from the baseline in self-efficacy on the SEQ-12 and Quitline use. We will also report on follow-up measurements of the Brief Questionnaire on Smoking Urge, NRT use, details of quit attempts, and smoker perceptions of the intervention. Attendance at follow-up events is encouraged by community health workers, and those who do not appear are offered the opportunity to complete the measures by telephone. In similar studies in this region, retention has been very high.

Throughout the 6-month participation period, we will collect additional assessment data through the assessment texts, including measures of abstinence assessments, and interest in and use of the Quitline. Quitline staff record details of intake assessment (number of calls, smoking status, and method used to quit if applicable) and fidelity of follow-up call completion. They also document referrals for the NRT. Data are entered into a secure data management system, with double-data entry occurring on a subset of participants to ensure accuracy or data entry.

### Adherence and Monitoring

Given that the intervention is low-risk, participants are unlikely to be withdrawn from the trial on the basis of safety concerns. Participants may withdraw at their own request. Participants are free to engage in other smoking cessation-related care and interventions during their participation. A number of actions are being taken to monitor and ensure adherence to intervention protocols. Research staff are trained on responsible conduct of the research. Consent materials are uploaded to a secure data capture system and reviewed by the project director. A sample of 20% (150/750) of enrollments are monitored by the site principal investigator, who then completes an adherence checklist. For the texting system, the programmer can monitor the texts being sent and received.

### Data Analysis

All primary analyses will be conducted on an intention-to-treat basis. However, secondary analyses will explore dose-response effects among those with variable levels of adherence to the intervention. All analyses will be two-sided, and the α error will be set at .05. We begin the statistical analysis by examining the univariate statistics and distributions. We will examine the balance of participant characteristics by study groups and account for any imbalances in our multivariable analysis. As appropriate, group differences will be tested using chi-square tests of independence, Z-test or *t* test (two-tailed), or the equivalent nonparametric tests depending on the distribution of the variables. In accordance with best practice, differences in baseline characteristics of the intervention and comparison groups will be established based on standardized differences rather than on tests of statistical significance [16,17].

The primary outcome was the patient tobacco cessation rate (quit rate) at 6 months follow-up visit and will be compared using a two-sided chi-square test. We will include a multivariable logistic regression model to adjust for any potential confounding factors, if needed. As a secondary outcome, we will compare self-efficacy between intervention and control conditions: means on the SEQ-12 (and its subscales) will be compared between the intervention and control groups over time using repeated-measures analysis of variance and Student *t* test. A generalized linear model for repeated measures will be used to adjust for potential confounding factors, if needed. There are no planned interim analyses, but they may be requested or approved by the Data Safety and Monitoring Board.

### Ethics and Confidentiality

The study has been approved by the institutional review board (IRB) at the UMMS (H00012953) and the Institute of Population, Health and Development, Vietnam (PHAD-2017/M2Q2-01). All important protocol modifications are approved by the IRB and registered with ClinicalTrials.gov. Research assistants provide a brief overview of the study, including the reason the study is being performed, a description of the study design, and the participant’s role in the investigation if he or she decides to participate. The participant is informed of the potential benefits of the study. The patient is also informed that the design of the study requires collection of personal identifiers of the safeguards in place to protect that information, and of his or her right to withdraw from the study at any time (at which point all personal identifier data collected will be destroyed). All participants provided written informed consent for study inclusion, administration of surveys, and carbon monoxide verification. At any time, participants may withdraw their consent.

All data are obtained by trained study staff and entered directly into electronic forms on a secure website designed for this purpose. We have created a texting service that is responsible for sending text messages, as well as receiving the assessment text responses (ratings and time to first quit). The software program we developed uses a secure application programmable interface to forward the patient’s phone number and send and receive the texts. The motivational messages do not contain any personal health information, and the texting service does not store the phone numbers (phone number data are not transferred to any site outside of Vietnam). Participants are informed of the potential risk of confidentiality of sending and receiving text messages during the informed consent process.

Participants are identified with a study number: the key is located centrally and securely and is accessible only to certified study personnel on an as-needed basis. All patient contact, including consent, interviews, and all study procedures with human subjects occurred onsite in Vietnam. All data are deidentified according to Health Insurance Portability and Accountability Act (HIPAA) standards before transfer to the investigators and staff for analytical purposes. Personal identifiers are abstracted onto separate confidential forms and are not transferred outside Vietnam. All records consisting of personal identifiers will be destroyed upon completion of the study. The data are stored in a HIPAA-compliant regulated environment and access will be only through a secure virtual private network. All the related identifiers of the participants are encrypted in the database.

### Safety and Adverse Events

As this trial is a phase 3 study including NRT and our behavioral intervention, we have established a Data and Safety Monitoring Board (DSMB). The DSMB is charged with reviewing protocols and consent documents for this trial, monitoring safety issues throughout the study, and the quality of the accumulating data, providing guidance on interim analyses and stopping rules. The DSMB comprises persons with no direct involvement in the study or conflict of interest with the research team conducting the randomized trial and has the authority to halt the trial as needed.

Participants who call the Quitline may receive over-the-counter NRT in the form of nicotine lozenges at 4-mg doses. The risk of NRT has been evaluated in detail: as this treatment has less nicotine than cigarettes, it is in general a risk reduction from active smoking. All participants who had a Food and Drug Administration contraindication to nicotine lozenge are excluded. Patients may become uncomfortable when asked about psychosocial factors such as smoking cravings or urges and are reminded repeatedly that they are under no obligation to respond to any question, and interviewers are trained and supervised in appropriate interview techniques. The principal investigator monitors all adverse events; all adverse events, both serious and nonserious, are summarized in the report to the IRB committee for annual study review and renewal.

Patients are given up to 6 weeks of NRT if requested during the study period, and free NRT is not maintained beyond the study period; participants revert back to usual care at their community health centers after the end of their involvement in the study. Participants in the comparison condition are allowed (but not actively encouraged or facilitated) to engage with the Quitline if they choose.

## Results

### Intervention Development and Adaptation

After existing candidate messages were professionally translated to Vietnamese, 14 consultants (former smokers who had used the Bach Mai Quitline services) and 7 professionals (5 counselors and 2 doctors from the Bach Mai Quitline) reviewed the 126 existing messages and were asked to identify messages that they liked and disliked. Reconciling the two sets of messages through a group review, there were 20 text messages that were disliked by none of the consultants or Quitline counselors, and these were identified for inclusion in the message pool. The message writing exercise with 10 former smokers led to the creation of 238 new smoker-written messages. After eliminating messages that were incomplete, unclear, or clearly redundant, a set of shortlisted messages was reviewed independently by researchers for preference and the top 44 messages were identified for inclusion in the message pool. Between the expert-written messages and the smoker-written messages, a total of 57 messages were selected for the intervention: seven messages to be sent during week 0 of the intervention, and two messages per week to be sent during weeks 1-25 of the intervention. The messages reflected several themes: calling the Quitline and Quitline as helpful, knowledge and health risks, self-efficacy and motivators, role of family, and quitting tips. The final messages were reviewed by a researcher who sequenced them using a social cognitive approach, for example, messages related to knowledge and health risks occurred earlier in the sequence and quitting tips occurred later in the sequence. The following are examples of motivational text messages received by the intervention group. The text messages were translated from Vietnamese and the original text can be found in Multimedia Appendix 1.

- “The following message was written by a smoker in your community...I felt like the counselors at the Bach Mai Quitline are close as family members, who want to help me to quit smoking.”
- “The following message was written by a tobacco cessation expert...Most people make repeated quit attempts before they are successful. You can succeed. Your doctor is available with treatment options and support to help you.”
- “The following message was written by a smoker in your community...Thinking about my family, my children, my grandchildren and people helped me stay focused on quitting.”

### Trial Status

The study was funded in August 2017. The study was reviewed and approved by the University of Massachusetts Medical School IRB in 2017. Recruitment began in November 2018. As of March 2021, 750 participants have been recruited from four communes, and a total of 700 (93.3%) participants completed the follow-up. An analysis of the trial results is in progress; results are expected to be published in late 2022.

## Discussion

### Summary

Mobile health Messaging to Motivate Quitline Use and Quitting (M2Q2) will examine whether a motivational text messaging intervention is effective in promoting cessation and treatment uptake among Vietnamese smokers. It is a unique opportunity to engage a challenging population of rural Vietnamese smokers in a novel intervention. This study examines the effectiveness of a culturally tailored text messaging intervention to motivate tobacco cessation and connect smokers to underused Quitline and NRT.

### Limitations

This study has several limitations. First, the study applies randomization at the individual level, allowing for ample power and a rigorous approach; however, it is possible that the close-knit nature of these rural communities causes participants to share information during the follow-up period, resulting in unintentional unblinding to allocation and contamination. Second, the global COVID-19 pandemic occurred during the trial, and Vietnam adopted stringent infection control measures in response. Recruitment was paused for a matter of weeks, but our original targets and retention rates did not appear to have been profoundly affected. COVID-19 rates were relatively low in Vietnam, but we will still test the potential confounding effect of the pandemic in our analyses, given the potential effect of COVID-19 on cessation rates in the comparison group. Finally, the study is limited to a moderate follow-up period of 6 months, rather than a longer follow-up period. In conclusion, this study examines the effectiveness of potential mobile health interventions that can be implemented nationwide in Vietnam and in other diverse populations that bear significant burdens from smoking.

### Dissemination Plan

We will present our work at relevant scientific conferences and publish our results in peer-reviewed literature. Manuscripts and other products arising from the study will be produced by the research team and will not involve professional writers. In line with guidelines from the International Committee of Medical Journal Editors, authorship will be contingent on the following criteria:

- Substantial contributions to conception and design, or acquisition, analysis, or interpretation of data
- Drafting of the study or critical revision for important intellectual content
- Final approval of the version to be published
- Agreement to be accountable for all aspects of the work in ensuring that questions related to the accuracy or integrity of any part of the article are appropriately investigated and resolved

In conclusion, mobile interventions for smoking cessation in low- and middle-income countries have been shown to be feasible and acceptable [18,19]. We anticipate that the culturally tailored M2Q2 intervention will promote tobacco cessation and treatment uptake in smokers in Vietnam.

## Appendix

Multimedia Appendix 1 Examples of motivational text messages received by the intervention group.

## Abbreviations

DSMB: Data and Safety Monitoring Board
IRB: institutional review board
NRT: nicotine replacement therapy
SEQ-12: 12-item Smoking Self-Efficacy Questionnaire
UMMS: University of Massachusetts Medical School
