# Supplementary material for: mHealth Messaging to Motivate Quitline Use and Quitting: Protocol for a Community-Based Randomized Controlled Trial in Rural Vietnam
Source: JMIR Res Protoc. 2021 Oct 7;10(10):e30947. doi: 10.2196/30947 (PMC8532014; doi:10.2196/30947)
Supplement: Multimedia Appendix 1 [file resprot_v10i10e30947_app1.pdf]

Examples of motivational text messages received by the intervention group

- "The following message was written by a smoker in your community...I felt like the counselors at the Bach Mai Quitline are close as family members, who want to help me to quit smoking." [Tin nhắn dưới đây là do một người hút thuốc tại cộng đồng của bạn viết...Tôi cảm thấy tư vấn viên tại Tổng đài cai thuốc thân tình như người nhà và muốn giúp tôi cai được thuốc.]

- "The following message was written by a tobacco cessation expert...Most people make repeated quit attempts before they are successful. You can succeed. Your doctor is available with treatment options and support to help you." [Tin nhắn dưới đây là do một chuyên gia về cai thuốc viết...Hầu hết mọi người phải cố gắng cai thuốc lá nhiều lần trước khi cai thuốc lá thành công. Bạn có thể thành công. Bác sĩ của bạn sẵn sàng đưa ra các phương pháp cai thuốc lá và giúp đỡ bạn.]

- "The following message was written by a smoker in your community...Thinking about my family, my children, my grandchildren and people helped me stay focused on quitting." [Tin nhắn dưới đây là do một người hút thuốc tại cộng đồng của bạn viết...Nghĩ về gia đình, con cháu và mọi người giúp tôi tập trung vào việc cai thuốc.]
